# Adenoid ameloblastoma of the mandible: report of a new case of a distinct type of odontogenic neoplasms

**DOI:** 10.1186/s12903-026-08711-x

**Published:** 2026-06-05

**Authors:** Nourhan Abou Madawi, Ahmed Abdou, Nermine El Bahey, Magi Moussa

**Affiliations:** 1 Oral Pathology Department, Faculty of Dentistry, Alexandria University, Champollion Street, Azarita, Alexandria, 21521 Egypt; 2 Maxillofacial and Plastic Surgery Department, Faculty of Dentistry, Alexandria University, Alexandria, 21521 Egypt; 3 Oral Pathology Department, Faculty of Dentistry, Pharos University, Alexandria, 21521 Egypt

**Keywords:** Odontogenic tumors, Adenoid ameloblastoma, Ameloblastoma, Adenomatoid odontogenic tumor, Case report

## Abstract

**Background:**

Adenoid ameloblastoma (AA) is a new type of odontogenic neoplasms added to the latest World Health Organization (WHO) classification. It has clinical course somewhat similar to ameloblastoma but yet more aggressive. Histopathologically, AA is formed of ameloblastomatous component arranged in a cribriform pattern, duct-like structures and whorled cellular condensations which make conventional ameloblastoma and adenomatoid odontogenic tumor in the main differential diagnosis. The clinical and radiographic features of AA are not characteristic which necessitate great care in histological examination. It is of paramount importance to distinguish between ameloblastoma with some glandular features and actual AA. Immunohistochemistry and/or molecular testing can provide definitive diagnosis of AA. Few cases of ameloblastoma with adenoid features were reported before considering AA as a separate entity. To the best of our knowledge, only two studies performed molecular testing and three studies used supporting immunohistochemical markers of AA.

**Case presentation:**

We report a case of a 19-year-old male patient who was presented with a painful swelling in the left side of the mandible for a period of four months. Past medical history and dental history were free. Radiographic examination showed multilocular radiolucency. Incisional biopsy revealed Ameloblastoma. Wide surgical excision was done. Microscopic examination of the surgical specimen showed histopathological features providing the diagnosis of AA. We further carried out immunohistochemistry to support the diagnosis. Intermaxillary fixation for a period of 4 weeks was carried out using wires for jaw stabilization and maintaining proper bite. The patient was followed up regularly every one month.

**Conclusions:**

Reporting of new cases of AA is of paramount importance for both surgeons and pathologists that increases their awareness about AA which is more aggressive than conventional ameloblastoma and thus, providing accurate management.

## Introduction

Adenoid ameloblastoma (AA) is a newly identified odontogenic epithelial neoplasm. Like conventional ameloblastoma, it is locally infiltrative and has an aggressive clinical behaviour [1]. It is considered as a separate entity from ameloblastoma in the fifth edition of the World Health Organization (WHO) classification of head and neck tumors [2]. Only few cases were reported in the literature. It tends to affect females more than males in their middle age. It often presents clinically as a painless swelling in the mandible with a high recurrence rate [1].

The histopathological features of AA are a mixture from the histology of conventional ameloblastoma, adenomatoid odontogenic tumor (AOT) and dentinogenic ghost cell tumor [DGCT] [3]. AA shows the arrangement of ameloblast-like cells and stellate reticulum-like cells as seen in conventional ameloblastoma in addition to cribriform arrangement. Duct-like structures and whorled masses (morules) are characteristic features. Dentinoid and clear cells are noted in the majority of cases and to a lesser extent ghost cells [2].

Both ameloblastoma and AOT exhibit mutations in MAPK signaling pathway. The majority of AOT cases harbor either KRAS p.G12V or p.G12R activating mutations [4]. BRAF acts downstream to KRAS in the MAPK signaling pathway. BRAF p.V600E mutation is a key mutational event in many cases of ameloblastoma [5].Molecular characterization of AA elucidated that it lacks BRAF and KRAS mutations supporting it as a distinct entity from conventional ameloblastoma and AOT. Moreover, Immunohistochemistry is a valuable diagnostic aid in pathology. It was found that it can help to distinguish AA from conventional ameloblastoma [3, 6].

We report a case of AA presented to our institution which adds to the limited cases reported in literature. We utilized immunohistochemistry in addition to recognizing the histopathologic features typical of AA to provide precise diagnosis. This can help improve our understanding and management of this new entity of odontogenic tumors.

## Case presentation

**Fig. 1:**
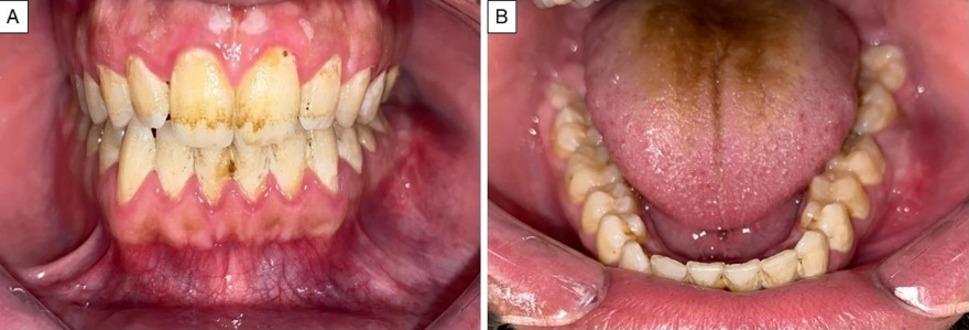
**A** and (**B**) Clinical intraoral presentation of the studied case of adenoid ameloblastoma

**Fig. 2:**
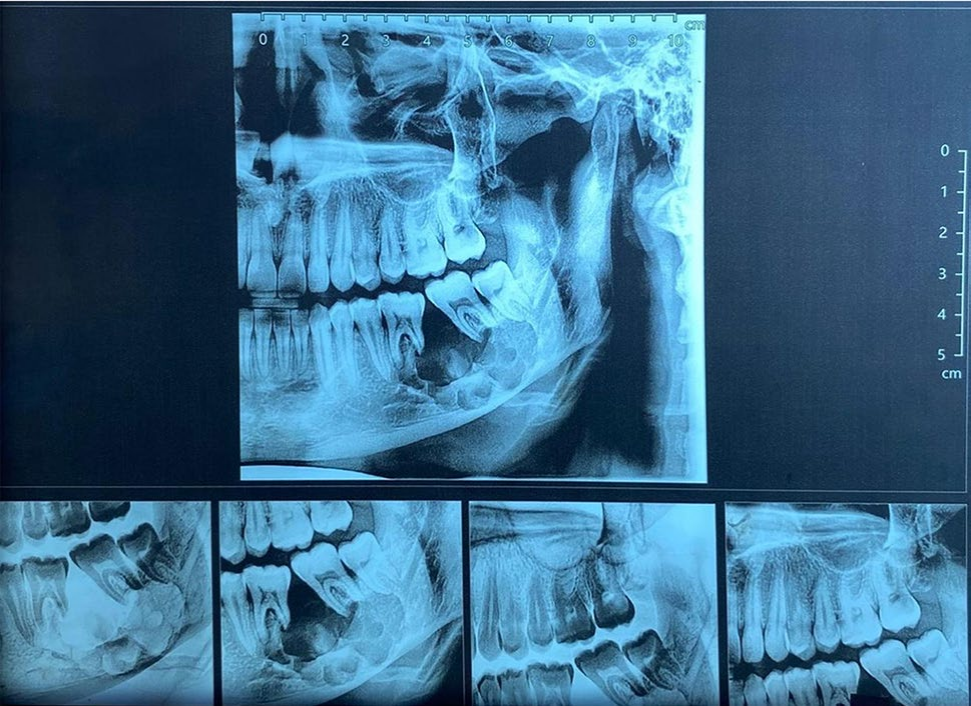
Segmented panoramic view showing multilocular radiolucency in the left side of the mandible related to molar teeth and causing root resorption

**Fig. 3:**
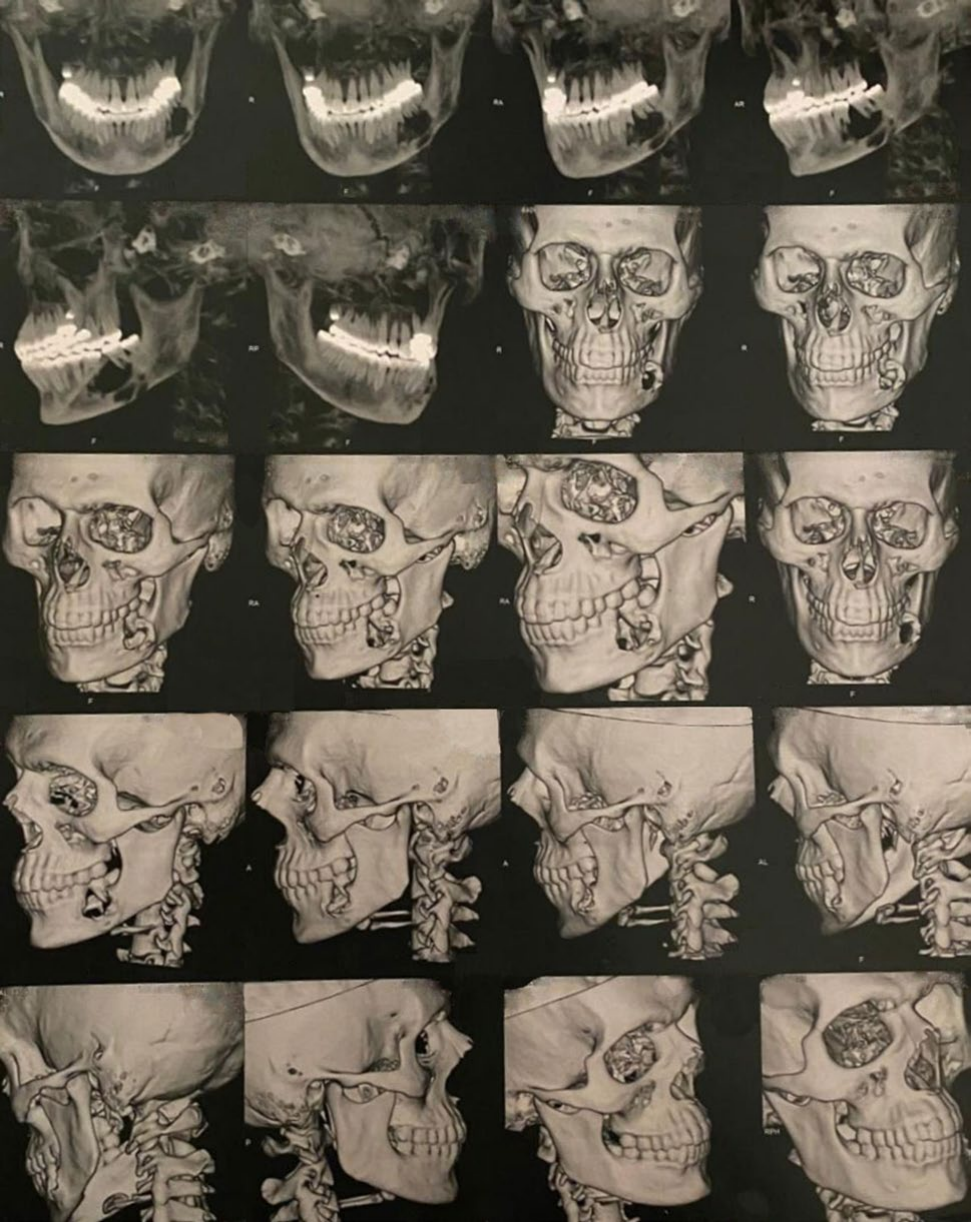
Cone beam computed tomography (CBCT) of the skull demonstrating the extent of bone destruction in the mandible

A 19-year-old male patient presented with a painful swelling in the left side of the mandible. The condition persisted for 4 months. Extraoral examination revealed swelling of the left cheek. Intraoral examination revealed swelling involving the left side of the mandible measuring approximately 2.5 cm x 2 cm related to molar teeth with buccal expansion but no perforation through the soft tissue was noted (Fig. 1). The patient’s medical and dental history was free. Panoramic x-ray showed honeycomb multilocular radiolucency related to lower left molar teeth (Fig. 2). Provisional diagnoses were ameloblastoma, odontogenic keratocyst, and aneurysmal bone cyst. Subsequently, Cone beam computed tomography (CBCT) was done revealing a destructive lesion in the left side of the mandible. There was root resorption of the first and second molar teeth (Fig. 3).

Incisional biopsy was taken and stored in 10% formalin solution. The specimen was processed and histopathological examination revealed proliferation of odontogenic epithelium in the form of islands within a fibrous tissue stroma showing inflammatory cell infiltrate. The islands are formed of peripheral ameloblast-like columnar cells with prominent palisading surrounding stellate reticulum-like cells. Diagnosis was ameloblastoma. Upon diagnosis, wide surgical excision was performed under general anaesthesia with extraction of lower left second premolar and first, second and third molar teeth. Grossly, the lesion measured about 3.5 × 2.5 × 1.5 cm. Intermaxillary fixation was carried out using wires for jaw stabilization and maintaining proper bite.

**Fig. 4:**
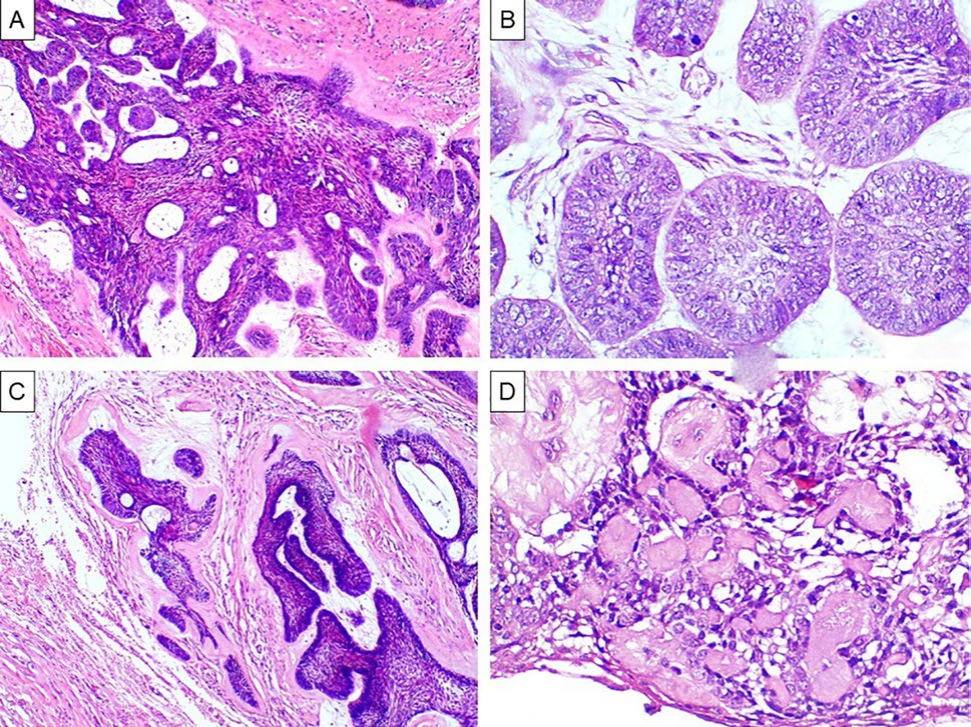
Histopathological findings characteristic of adenoid ameloblastoma (**A**) cribriform arrangement of ameloblast-like cells and stellate reticulum like cells, duct like structures and morules (H&E, magnification x100) (**B**) Multilayered-basal cells with transition to round or ovoid morphology (H&E, magnification x400) (**C**) Dentinoid (H&E, magnification x100) (**D**) Ghost cells and clear cells (H&E, magnification x400)

Laboratory processing of the surgical specimen was done. Haematoxylin and eosin-stained slides were thoroughly examined by experienced pathologists. Tissue sections showed proliferation of odontogenic epithelium in the form of peripheral ameloblast-like cells with reversed polarity and central stellate reticulum-like cells. Interlacing network forming cribriform pattern was evident with duct-like structures filled with myxoid secretion as well as morules (Fig. 4A). The basal cells were multi-layered with transition to round or ovoid morphology with vesicular nuclei. In some areas, basaloid pattern was evident. (Fig. 4B). Cystic changes were also detected. Varying amount of dentinoid was found (Fig. 4C). Aberrant keratinizations reminiscent of ghost cells were noted and were surrounded by clear cells (Fig. 4D). These histopathological features suggested diagnosis of AA.

**Fig. 5:**
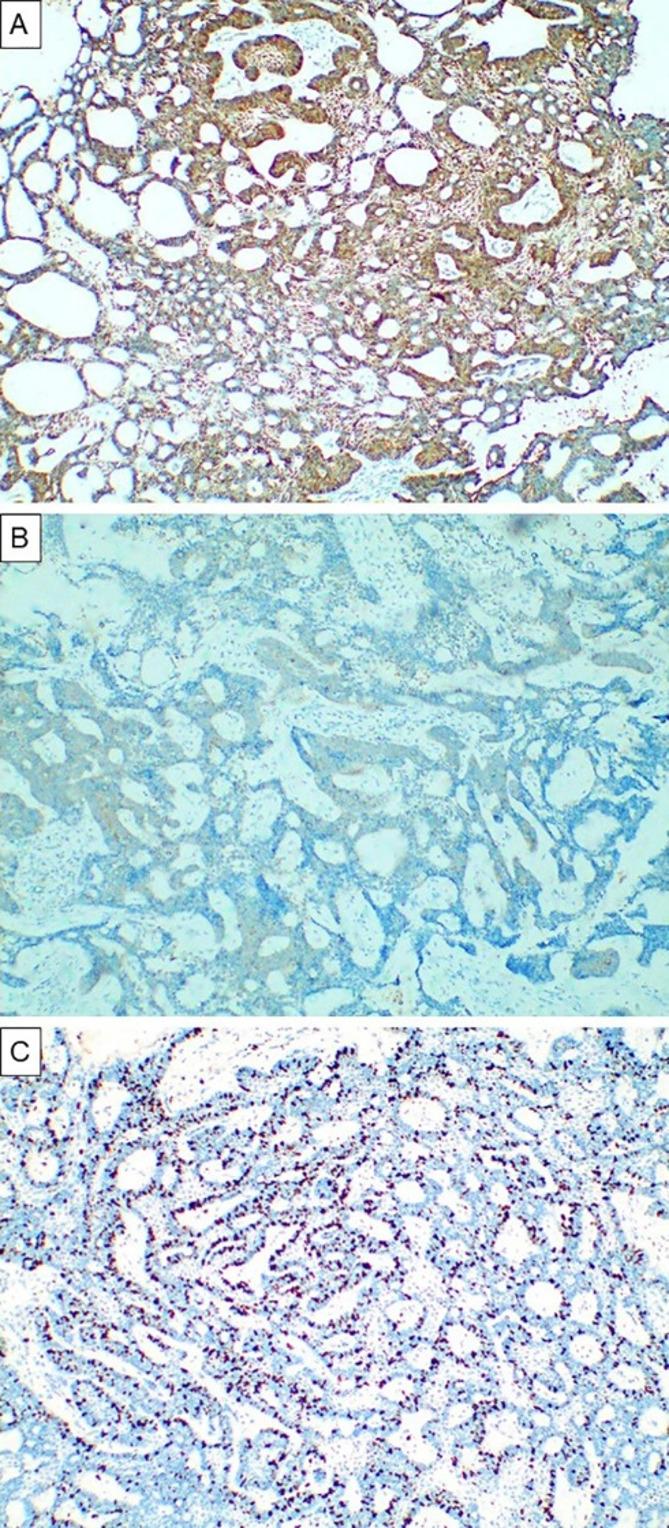
Immunohistochemical findings. Adenoid ameloblastoma showing (**A**) strong immune reaction to beta-catenin (magnification x100) (**B**) faint immune reaction to BRAF (magnification x100) (**C**) high Ki-67 score (magnification x100)

To confirm the diagnosis, immunohistochemical staining by BRAF, beta-catenin and Ki-67 was done, the tumor cells showed strong beta-catenin expression (Fig. 5A) while they showed faint positive reaction to BRAF (Fig. 5B). The Ki-67 score was 15 (Fig. 5C).

**Fig. 6:**
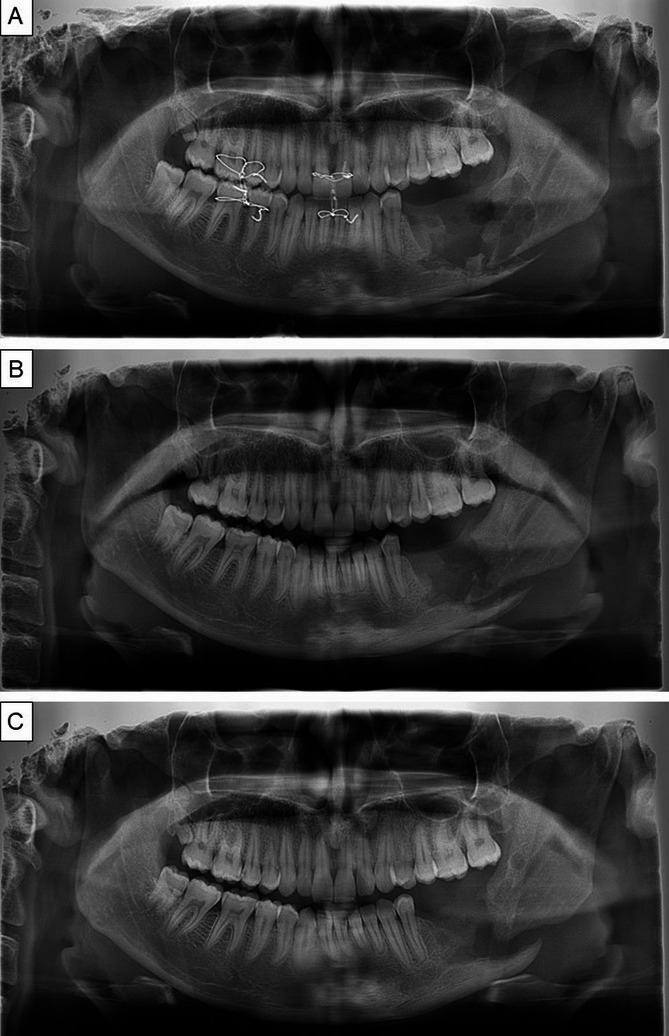
Post-operative panoramic x ray after (**A**) one week (**B**) one month (**C**) 6 months

The patient was periodically followed up every month. At first, there was limited mouth opening which subsided over time as noted in subsequent visits. The surgical site was healing with bone formation. There was no evidence of recurrence after 6 months (Fig. 6A-C). The patient was satisfied with the outcome.

This study was approved by the Research Ethics Review Committee of Faculty of Dentistry Alexandria University (IRB NO: 00010556-IORG 0008839). Ethics Committee No:1217-12/2025.

## Discussion and conclusions

Conventional ameloblastoma is characterized by various histopathologic patterns. The most common patterns are follicular and plexiform. Less common patterns are acanthomatous, granular cell, desmoplastic, and basal cell [7]. In some cases, there is overlap of patterns and it is difficult to categorize cases as a definite subtype [8]. Yet, these patterns barely affect the clinico-radiographic behaviour of the tumor [7].

It is noteworthy that the odontogenic epithelium has a variable differentiation potential. Proliferation areas mimicking ameloblastoma, AOT, ameloblastic fibroma, ameloblastic fibroodontoma, and calcifying epithelial odontogenic tumor were depicted in occasional jaw lesions which were given the name hybrid or collision tumors. However, the biological behaviour of these tumors is poorly determined [9]. In 1994, Brannon [10] recognized and studied a lesion formed of ameloblastomatous differentiation with clear cells exhibiting AOT-like proliferation as well as dentinoid and gave it the term adenoid ameloblastoma with dentinoid [10]. Due to its similar clinical course to ameloblastoma, it was considered as one subtype of it [11, 12]. Other terms were used such as plexiform ameloblastoma with dentinoid and atypical plexiform ameloblastoma with dentinoid. It is known that inductive changes are not common in ameloblastoma suggesting that dentinoid is a product of the supporting stroma not a neoplastic element [13]. The term adenoid ameloblastoma (AA) was preferred by some authors due to lack of dentinoid in some of their studied cases [6, 9]. Interestingly, it was found that AA lacks BRAF and KRAS mutations which are present in conventional ameloblastoma and AOT respectively [14]. On the other hand, Bilodeau and Seethala [6] reported positive immunohistochemical expression of beta catenin in their studied cases [6]. Also, Bastos et al. [3] found that AA expresses beta catenin which is linked to tumors with ghost cells such as pilomatrixoma. They performed both Sanger sequencing and immunohistochemistry [3]. This resulted in the involvement of AA in the recent WHO classification of maxillofacial and bone tumors as a separate entity from conventional ameloblastoma and designated as adenoid ameloblastoma [2].

The current case affected a young adult male which is in accordance with the reported demographic findings of AA [2]. AA occurs more commonly in the mandible especially the posterior region [15] which was also noted in our case. Furthermore, the patient complained of pain which is an uncommon clinical feature reported in minority of cases [9].

Radiographically, AA presents with radiolucency either unilocular or multilocular. Cortical perforation and root resorption are possible findings [2, 16]. In our case, there was slight tooth displacement with resorption of roots.

Histologically, the ameloblastomatous component of AA is arranged in the form of plexiform or follicular pattern. The majority of the reported cases showed plexiform pattern. Acanthomatous pattern can be noted (15). Mixed pattern (follicular, plexiform, granular, desmoplastic) is also encountered (16). Our case showed mainly plexiform pattern. In some areas, basaloid pattern was evident which was not reported before.

AOT is a benign odontogenic tumor that is considered as hamartoma. Clinically, it occurs more common in females in the second and third decades of life. It has a propensity to affect the anterior maxilla. AOT has a benign course with no cortical perforation or recurrence. Thus, AA clinically differs from AOT with more resemblance to conventional ameloblastoma. Common histopathological features between AA and AOT include duct-like structures and morules. The most important histological difference is the presence of fibrous tissue capsule in AOT while AA is locally invasive [16].

DGCT is a rare odontogenic tumor that is benign but locally infiltrative. It is considered as a solid variant of calcifying odontogenic cyst (COC) [17]. It is characterized histologically by islands and sheets of odontogenic epithelium reminiscent of ameloblastoma, ghost cells of variable amount and dentinoid that often encloses individual or groups of epithelial cells and ghost cells. Mineralization of dentinoid is common and mineralization also originates from ghost cells but to a lesser extent. AA differs from DGCT in that DGCT does not exhibit AOT-like configuration. Also, ghost cells and dentinoid are inherent histopathological features of DGCT, while they are not always present in AA. Radiographically, DGCT also differs from AA in that DGCT appears mixed radiolucent radiopaque while AA appears radiolucent. Another tumor which has ghost cells and dentinoid and should be differentiated from AA is ghost cell odontogenic carcinoma (GCOC). GCOC shows malignant criteria such as nuclear and cellular pleomorphism, loss of ameloblastomatous differentiation with increasing grade and areas of necrosis. These features are not present in AA [2]. COC, DGCT and GCOC lack the adenoid differentiation illustrated by duct-like structures. Duct-like structures is a common feature between AOT and AA, however, the other distinctive histopathological features of AA should differentiate it from AOT [2]. Moreover, cases previously diagnosed as aggressive AOT should be re-evaluated as they could probably represent the new entity AA.

Hypercellularity is a feature of AA as detected in the studies of Loyola et al. [9] and Jayasooriya et al. [16]. Our case also exhibited increased cellularity in accordance with previous reports. Mitosis may be noted in AA [15]. Meticulous histological examination is mandatory to exclude ameloblastic carcinoma which is a malignant neoplasm showing malignant criteria, loss of ameloblastomatous differentiation and occasional vascular or perineural invasion but no characteristic findings of AA like dentinoid [9, 16]. Another proposed tumor that was considered in the differential diagnosis with AA is odontogenic carcinoma with dentinoid. Odontogenic carcinoma with dentinoid exhibits clear cells, cellular pleomorphism, mitotic activity and dentinoid lay down [14]. But due to its overlapping features with other odontogenic carcinomas such as GCOC and clear cell odontogenic carcinoma and the lack of molecular characterization, it was omitted from the latest WHO classification [2].

Molecular analysis of tumors has dramatically helped in distinguishing and categorizing tumors into definite entities which has a great impact on accurate diagnosis and proper management of patients [2]. The study of Coura et al. [14] in 2021 has put the basis for considering AA as a distinct type of odontogenic neoplasms and not as a subtype of conventional ameloblastoma and also negating its diagnosis as AOT. They carried out TaqMan allele-specific qPCR to detect BRAF mutation characteristic of conventional ameloblastoma and also KRAS mutation characteristic of AOT. None of the cases exhibited these mutations [14]. In the present study, we could not perform TaqMan allele-specific qPCR as it is unavailable. We performed immunohistochemistry. Most of the tumor cells were negative for BRAF. On the other hand, the tumor cells were strongly positive for beta-catenin consistent with the findings of Bastos et al. [3] which advocated the unique nature of AA [3]. Similarly, a case reported by Haraguchi et al. in 2025 revealed negative BRAF expression and positive beta-catenin expression in accordance with our findings [18].

Ki-67 protein is a proliferation marker with clinical significance that is widely used on different types of human tumors to assess proliferation of cells and subsequently grading of tumors [19]. It has been considered as a good prognostic marker in many studies [20–22]. Before the latest WHO classification, Loyola et al. [9] reported 5 cases of ameloblastoma with glandular features and dentionoid which exhibited higher Ki-67 proliferation score in comparison to conventional ameloblastoma and was approximating that of ameloblastic carcinoma. Increased mitotic activity was detected. They suggested that those cases should be clinically distinguished due to their aggressive behaviour and recurrence incidence. High Ki-67 labelling index reflects the aggressive nature, local infiltration and recurrences of AA [9]. A study was conducted to compare between ameloblastoma and ameloblastic carcinoma. Ki-67 proliferative index for ameloblastoma ranged from to 0.35% to 6.89% while for ameloblastic carcinoma, it ranged from 11.87% to 53.29%. In our case, Ki-67 score was 15% which was similar to that of ameloblastic carcinoma [23].

Our case was treated by wide surgical excision which is the typical treatment but radiotherapy is another treatment modality that can be adopted in case of extensive maxillary tumors or recurrence [9, 24]. Recurrence of AA may be attributed to misdiagnosis of some cases as AOT which resulted in conservative treatment [9]. A previous case of AA was misdiagnosed primarily as AOT which recurred three times that raised suspicion of incorrect diagnosis and great care to reconsider diagnosis. It turned out to be AA [11]. Recurrence of maxillary AA could be due to difficulty in performing complete excision with free margins due to porous nature of the maxilla [24]. 

In addition to BRAF mutation, Ameloblastoma also harbors other mutations like SMO [15]. This calls for more study of AA cases with attention to their molecular profile to confirm or contradict the distinct nature of AA. Staining with specific immunohistochemical markers (BRAF, beta-catenin) is also recommended in future studies to support current results.

The limitation of this study is lack of availability of molecular testing.

In conclusion, AA is a rare new entity of odontogenic tumors that shares some histopathological features with conventional ameloblastoma and AOT, yet exhibits different clinical behaviour. Meticulous care is essential to correctly diagnose cases for optimum selection of treatment modality and better prognosis.

## Data Availability

All data generated or analysed during this study are included in this published article.

## Abbreviations

AA: Adenoid ameloblastoma
AOT: Adenomatoid odontogenic tumor
CBCT: Cone beam computed tomography
COC: Calcifying odontogenic cyst
DGCT: Dentinogenic ghost cell tumor
GCOC: Ghost cell odontogenic carcinoma
WHO: World Health Organization
